# Biatrial enlargement as a predictor for reablation of atrial fibrillation

**DOI:** 10.7150/ijms.47568

**Published:** 2020-10-18

**Authors:** Qiang Kong, Lisheng Shi, Ronghui Yu, Deyong Long, Yucong Zhang, Yujia Chen, Jing Li

**Affiliations:** 1 Division of cardiology, Capital Medical University Xuanwu Hospital, No. 45 Changchun Street, Xicheng District, Beijing 100053, PR China; 2 Division of cardiology, Beijing Anzhen Hospital, Capital Medical University, No. 2 Anzhen Road, Chaoyang District, Beijing 100029, PR China.

**Keywords:** Atrial fibrillation, Catheter ablation, Biatrial enlargement

## Abstract

**Purpose**: We aimed to determine whether biatrial enlargement could predict reablation of atrial fibrillation after first ablation.

**Methods:** 519 consecutive patients with drug resistant atrial fibrillation [paroxysmal AF (PAF) 361, non-PAF 158] who underwent catheter ablation in Capital Medical University Xuanwu hospital between 2009 and 2014 were enrolled. Biatrial enlargement (BAE) was diagnosed according to trans-thoracic echocardiography (TTE). Ablation strategies included complete pulmonary vein isolation (PVI) in all patients and additional linear ablation across mitral isthmus, left atrium roof, left atrium bottom and tricuspid isthmus, or electrical cardioversion on the cases that AF could not be terminated by PVI. Anti-arrhythmic drugs or cardioversion were used to control the recurred atrial arrhythmia in patients with recurrence of atrial fibrillation after ablation. Reablation was advised when the drugs were resistant or that patient could not tolerate. Risk factors for reablation were analyzed.

**Results:** After 33.11±21.45months, 170 patients recurred atrial arrhythmia, and reablation were applied in 117 patients. Multivariate Cox regression analysis demonstrated that that biatrial enlargement (BAE, HR 1.755, 95%CI 1.153-2.670, P=0.009) was an independent predictor for reablation and was associated with reablation (Log rank P=0.007).

**Conclusion:** Biatrial enlargement is an independent risk predictor for the reablation in atrial fibrillation patients after first ablation.

## Introduction

Atrial fibrillation (AF) is the most common sustained cardiac arrhythmia 1. Catheter ablation of pulmonary veins or left atrium have been proved to be superior to anti-arrhythmic therapy in reducing AF recurrence and maintaining sinus rhythm 3,4. However, pulmonary vein isolation (PVI) or PVI plus additional linear ablation is associated with a considerable recurrence rate 5. Despite that anti-arrhythmic drugs or cardioversion were used to control the recurred atrial arrhythmia, almost 10% patients had to accept reablation due to atrial arrhythmias that refractory to anti-arrhythmic drugs 6. Risk factors have been identified to predict the recurrence of atrial fibrillation after ablation, such as left atrial enlargement (LAE), right atrium diameter (RAD), atrial tissue fibrosis, and low-voltage areas detected by high-density electroanatomical mapping in left atrium 7,8,9,10,11. However, risk factors to predict the reablation in patients with recurrence of atrial fibrillation are still uncertain. Recent study 12 had found that biatrial volume were independent predictors of AF recurrence after PVI. However, the predictive value of atrial enlargement of reablation in patients with recurrence of AF is still unknown. In this study, we assessed biatrial enlargement as a predictor for reablation in atrial fibrillation patients.

## Methods

### Study population

All procedures were in accordance with the ethical standards of the responsible committee on human experimentation and with the Helsinki Declaration of 1964, which was revised in 2013. All experimental protocols were approved by the Institutional Review Boards of the Capital Medical University Xuanwu hospital. All participants provided written informed consent.

This was a retrospective study. We reviewed 519 patients undergoing their primary catheter ablation of symptomatic drug resistant AF (paroxysmal AF n=361; non-paroxysmal AF n=158) in the heart center of Capital Medical university Xuanwu hospital from January 2009 to December 2014. Patients with chronic pulmonary disease, pulmonary hypertension, right-sided heart disease such as Ebstein's anomaly, and arrhythmogenic right ventricular cardiomyopathy (ARVC) were excluded. Informed consent for the AF ablation procedure was obtained from patients.

### Echocardiography

**Figure 1:**
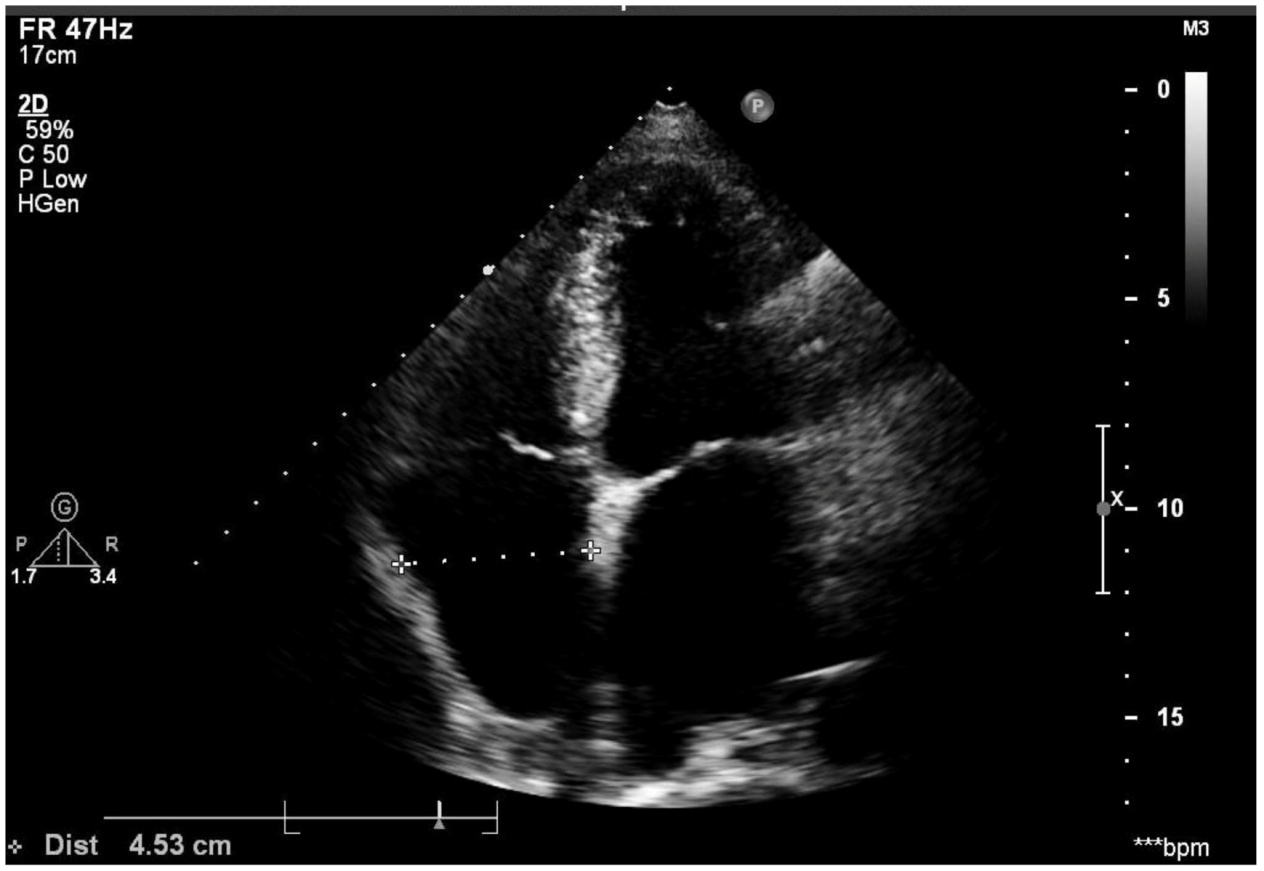
Right atrium diameter was measured using the four-chamber apical view during the systolic phase and left atrium diameter was measured also on this image.

All the patients underwent trans-thoracic echocardiography (TTE) and trans-esophageal echocardiography (TEE) (Vivid, GE). The Left and right diameter was measured on the four-chamber apical view during systolic phase. The horizontal diameter of left atrium (LA) was determined as the measurement from the middle of mitral isthmus to the LA roof (endocardial surface), while the horizontal diameter of right atrium (RA) was determined as the measurement from the middle of tricuspid isthmus to the RA roof (endocardial surface), see Figure 1. LA and left atrial appendage (LAA) thrombus was excluded by TEE. Our standard techniques for echocardiography examination also included that all measurements were averaged from at least three cardiac cycles and then reviewed by two echocardiologists. Right atrium enlargement (RAE) was diagnosed from that the horizontal diameter of right atrium was above 40mm regardless of sex. Left atrium enlargement (LAE) was diagnosed that left atrium horizontal diameter was above 40mm for male and 35mm for female. Biatrial enlargement (BAE) was diagnosed when a patient was with both LAE and RAE. The normal value of echocardiography measurements in the Chinese population referred to the consensus statements endorsed by the Beijing Task Force on Echocardiography 13.

### Electrophysiology study and AF radiofrequency ablation

Antiarrhythmic drugs (AADs) were discontinued for at least five half-lives before ablation. Oral anticoagulation (warfarin) was discontinued 3 days prior to the procedure, and low molecular weight heparin was administered for bridging. The procedure was performed with patients under conscious sedation. First, a decapolar catheter was positioned in the coronary sinus through left subclavian vein. Then transseptal puncture was accomplished with Sanjude transseptal puncture needle (St. Jude Medical Inc.). Following completion of transseptal access, a bolus of unfractionated heparin is given (50 U/kg body weight) and repeated for procedures lasting longer than 4 h or if the ACT falls below 200 s. After that, a 3.5-mm irrigated ablation catheter (Biosense-Webster Inc, Diamond Bar, CA, USA) was advanced to the LA for mapping and ablation. Mapping and ablation were guided by a 3D electro-anatomical mapping system (CARTO, Biosense-Webster Inc.) or non-contact mapping system (Ensite NavX system, St. Jude Medical Inc). The sheath was continuously perfused with heparinized saline at 2-3 ml/h.

Circumferential pulmonary vein isolation (PVI) was carried out for paroxysmal atrial fibrillation (PAF) patients. For persistent or long-standing persistent AF patients, additional linear ablation across LA roof, mitral isthmus, LA bottom and tricuspid isthmus were carried out besides PVI. Procedure endpoint was PVI for PAF patients and complete block of the lines besides PVI for persistent or long-standing persistent AF cases. Electrical cardioversion was applied if the atrial fibrillation could not terminated by PVI plus additional linear ablation. The specific definitions to PVI and linear block were detailed in previous report 14.

### Follow up

After discharge, patients were followed by their referring cardiologist. During the first three postoperative months, anti-arrhythmic drugs such as amiodarone or propafenone or sotalol were used for every patient. Patients with AF recurrences which were drug resistant were advised to undergo electrical cardioversions within 3 months after ablation (blanking period). A 12-lead ECG was obtained at 3, 6, and 12 months in all patients. Patients with no recurrence and a CHADS-VASC score <2 stopped taking warfarin 3 months following the procedure. Anti-arrhythmic drug was stopped 3 months after ablation if a patient had no recurrence of atrial tachyarrhythmia. Recurrence of AF was defined as AF or atrial tachycardia lasting >30 seconds after blanking period. Anti-arrhythmic drugs were used for recurrence of atrial arrhythmia after blanking period. Reablation was advised when the drugs were resistant or that patient could not tolerate. Anticoagulation strategy was strictly carried out according to CHADS-VASC grading system for each patient.

### Statistical analysis

For baseline characteristics, the Kolmogorov-Smirnov test was used to test the normality of distribution. Continuous variables are shown as mean ± standard deviation (SD) and compared using a two-tailed Student t test, while medians (Q1, Q3) and Mann-Whitney U tests were used for non-normally distributed variables. Categorical data are reported as counts and percentage (%) and between-group comparisons were made using the Pearson Chi-square or Fisher exact test. Variables that were statistically significant in univariate regression models (P value <0.1) were included in multivariate binary logistic regression model using a “forward conditional” method to determine whether they remained significant after adjustment for potential confounders. Risk factors associated with reablation were determined by a Cox regression model. All tests were two sided and P values <0.05 were considered statistically significant. Analyses were performed by IBM SPSS 25 (SPSS, IBM, Armonk, NY, USA).

## Results

### Demographic characteristics of study population

As in the Table 1, Patients with PAF (n=361) had smaller LA and RA diameter, smaller LVEDD and cardiac output, higher LVEF and less heart failure compared with non-PAF individuals (n=158). However, age, sex, hypertension, diabetes mellitus, CHADS2 sore and CHADS-VASC score were similar.

### The overall procedural outcome

According to right atrium diameter and left atrium diameter, patients were divided into two groups, Biatrial enlargement group (BAE, n=100) and non-Biatrial enlargement group (Non-BAE, n=419). The overall procedural outcome and clinical characters were listed in the Table 2. As in the Table 2, the procedure time was longer, and the proportion of persistent AF, PVI plus additional linear ablation, electrical cardioversion, recurrence and reablation were higher in BAE group.

After an average of 33.11±21.45 months, atrial arrhythmias recurred in 170 patients, and could be well controlled by anti-arrhythmic drugs in 53 patients. However, for those that were refractory to anti-arrhythmic drugs or those that could not bear the side effects of anti-arrhythmic drugs, reablation was applied in the rest 117 patients. The difference between two groups was listed in Table 3. As in Table 3, the age was older, the CHADS-VASC scale was higher and the proportion of CAD was lower among the patients that recurrent AF could be controlled by drugs.

### Association of reablation with clinical characters

The correlation between reablation and persistent AF (r=0.114, P=0.009), left atrium diameter (r=0.150, P=0.001), BAE (r=0.122, P=0.005) were moderate. Reablation was also correlated with mitral E peek (r=0.092, P=0.036) and right atrium diameter (r=0.092, P=0.036, as in Table 3).

**Figure 2:**
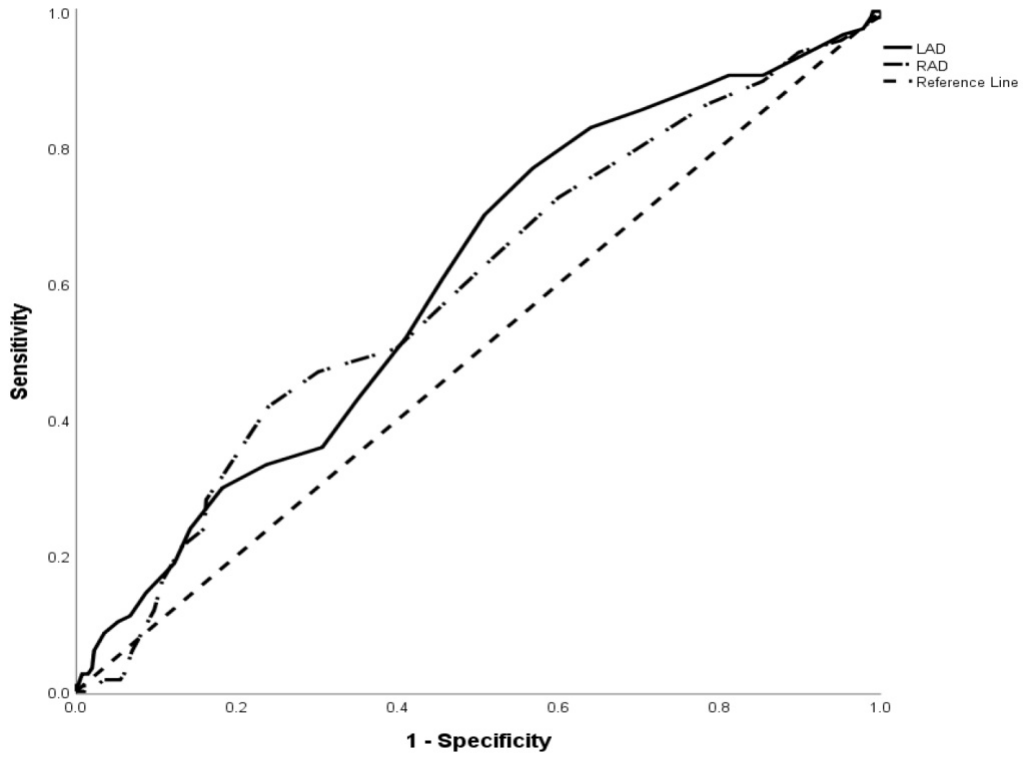
Receiver operating characteristic (ROC) curve showed the ability of RAD, LAD in predicting reablation in atrial fibrillation patients. A cutoff value of LAD 38.5 mm was associated with an area under the curve (AUC) of 0.603 and standard deviation of 0.029 (95% CI 0.546-0.660, P = 0.001). The cutoff value of RAD 38.5mm was associated with area under the curve (AUC) of 0.589 and standard deviation of 0.030 (95%CI 0.531-0.648, P=0.003).

Receiver operating characteristic (ROC) curves were built to establish the values that represented the cutoff point of right atrium diameter (RAD) and left atrium diameter (LAD) with the greatest sensitivity and specificity to predict reablation (as Figure 2). A cutoff value of LAD 38.5 mm was associated with an area under the curve (AUC) of 0.603 and standard deviation of 0.029 (95% CI 0.546-0.660, P = 0.001). The cutoff value of RAD 38.5mm was associated with area under the curve (AUC) of 0.589 and standard deviation of 0.030 (95%CI 0.531-0.648, P=0.003).

**Figure 3:**
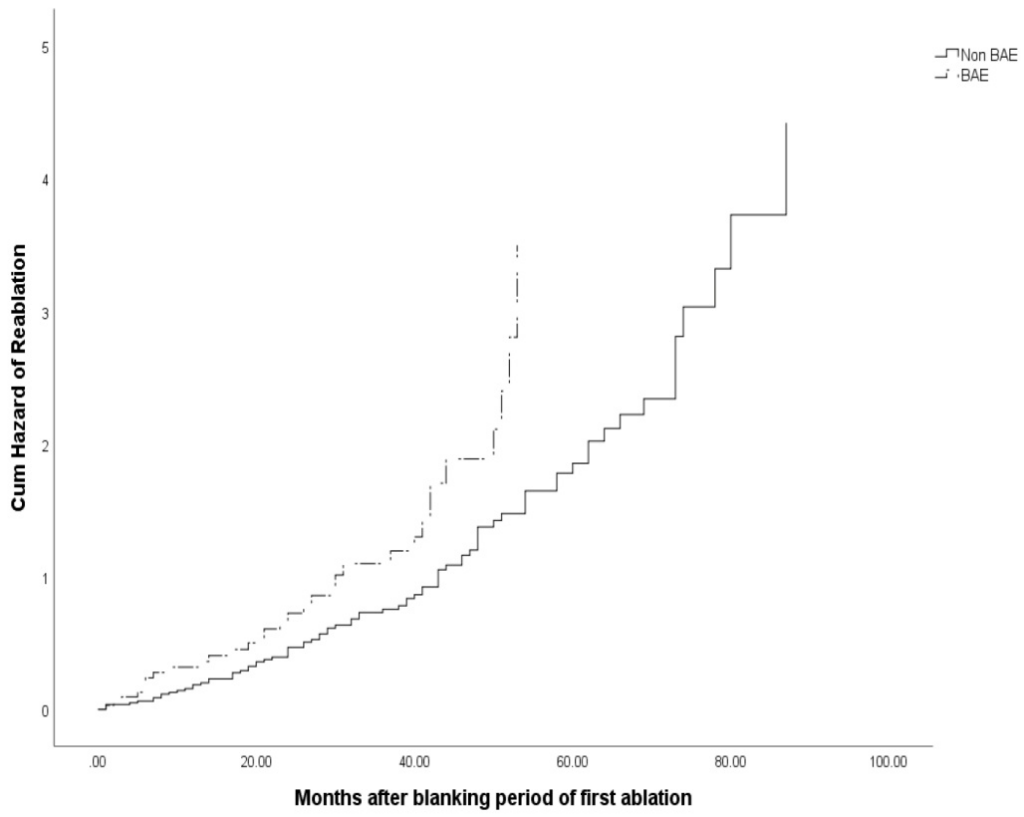
Cum hazard of reablation of patients with BAE or Non-BAE.

All the following confounders that potentially might have effect on procedural outcome based on prior knowledge or expected clinical relevance were entered into a Cox regression model. The variables were gender, age, persistent AF, hypertension, diabetes, coronary artery disease, LVEF, EDV, BAE, Mitral E peak. Multivariate Cox regression analysis demonstrated that BAE (HR=1.755, 95%CI 1.153-2.670, P=0.009) was the only independent predictor for reablation, as in Table 5. Kaplan-Meier survival analysis curve showed significant difference of cum hazard of reablation between those with BAE and Non-BAE (log-rank P = 0.007, Figure 3).

### Subgroup analysis based on biatrial enlargement (BAE) in patients with left atrium enlargement

In order to determine the effects of left atrium diameter on the reablation, we carried out subgroup analysis based on BAE (100/519) in patients with LAE (287/519). We found that procedure time, left atrium diameter, LVEF, heart failure, recurrence, reablation were different significantly between LAE only and BAE patients. See Table 6.

## Discussion

As we know, anti-arrhythmic drugs or cardioversion were used to control the recurred atrial arrhythmias for the patients with recurrence of AF after ablation. In our data, atrial arrhythmias recurred in 170 patients, and could be well controlled by anti-arrhythmic drugs in 53 patients. We compared the difference of the clinical characters between the patients with drugs and the patients underwent reablation to control the recurrent AF, and found out that the patients with drugs were older, the CHADS-VASC scale of them was higher and the proportion of coronary artery disease was lower.

Recent studies 15, 16 had demonstrated that right atrium remodeling was associated with atrial fibrillation and right atrium structure and functions were closely associated with AF development. In our research, we found that the process duration was longer and the proportion of PVI plus additional lines was higher for patients with BAE. For BAE patients in our data, the proportion of persistent atrial fibrillation was higher and left atrium diameter was larger. Since left atrium remodeling was associated with atrial fibrillation and increased atrial volume, interstitial fibrosis, and increased myocardial stretch favor the sustainability of atrial fibrillation 17. Thus, we could suppose that atrial fibrosis in BAE patients might be more severe than that in Non-BAE patients. Maybe that was why longer process time and PVI plus additional lines were common for BAE patients in first ablation.

Left atrium size and right atrium diameter were found to be associated with recurrence of AF after catheter ablation 7, 8, 9, 18. In our study, there were 170 patients that recurred atrial arrhythmias, including paroxysmal AF, atrial flutter, atiral tachycardia. Some of them could be controlled by anti-arrhythmic drugs, and for these patients, anticoagulation strategy was strictly carried out according to CHADS-VASC grading system. In the reablation, we found that ablation of pulmonary veins and lines in left atrium could terminate most of recurred atrial arrhythmias. Thus, gaps in pulmonary veins and incomplete block of left atrium ablation lines led to the recurrence of atrial arrhythmias in most patients in our data.

Although redo AF ablation was substantially more effective than AAD in reducing the progression and prevalence of AF after the failure of an initial ablation 19, it was not everyone who had recurred AF after catheter ablation that needed reablation, especially for those patients that atrial arrhythmia could be well controlled by anti-arrhythmic drugs. Recent multicenter clinical research in Europe had demonstrated that 9% of patients received a repeat ablation after PVI and 11% of patients were re-ablated after PVI plus additional lines or CFAE in left atrium. The proportion of the use of anti-arrhythmic drugs was decreased after first ablation and almost 32%-34% of patients used anti-arrhythmic drugs at 12 months follow up 6. In our data, almost 70% (117/170) of patients with recurred atrial arrhythmia underwent reablation. The reason lay in that our follow up time was longer (33.11±21.45months VS 12 months) and the constituent ratio of the patients were also different.

Receiver operating characteristic (ROC) curves was built to establish the values that represented the cutoff point of RAD and LAD to predict reablation. We found the cutoff values of LAD and RAD were both 38.5 mm. According to TTE, LAE was diagnosed when LAD was above 40mm for male and 35mm for female, while RAE was diagnosed from that RAD was above 40mm regardless of sex. From ROC curve, we knew that the sensitivity of prediction of reablation for LAD 35mm (diagnose of LAE for female) was 88.9% and the specificity was 22.4%. While for male, the sensitivity of the LAD 40mm (diagnose of LAE for male) was 60.7% and the specificity was 54.5%. However, the sensitivity of prediction of reablation for RAD 40mm (diagnose of RAE regardless of sex) was 28.2% and specificity was 83.8%. Since the AUC was not so large (only 0.603 for LAD), the strength of prediction power for reablation for diameter of atrium was only moderate.

As left atrium enlargement was independent predictor for occurrence of AF after first or repeat ablation 8, 18, we did the subgroup analysis based on the BAE in LAE patients to evaluate the effects of left atrium diameter on the reablation. We found that left ventricular ejection fraction was decreased (BAE 63(55, 68) VS LAE-only 66(60, 70) P=0.001), and heart failure were more common (BAE 18/100 VS LAE-only 9/187, P=0.000), which meant that left ventricular systolic function was even worse for BAE patients. Also, persistent atrial fibrillation, additional lines ablation besides PVI and electrical cardioversion were more common among these patients in the first ablation process. Multivariate Cox regression analysis showed that BAE (HR 1.620, 95%CI 1.020-2.574, P=0.041) was an independent predictor for reablation among the patients with enlarged left atrium. Kaplan-Meier survival analysis demonstrated that BAE was also associated with more reablation (Log rank P=0.026) in these patients.

## Limitations

This was retrospective study and was only a single institutional study. For criteria of reablation, we had to take the patients' desire into consideration, thus subjective factors would affect the result. In our data, reporting and selection bias could not be fully excluded. All comparisons between groups have to be interpreted with caution because PVI plus additional lines may have been performed more frequently in patients with more extensive substrate who would have had an even poorer prognosis with a PVI approach. Due to the non-standardized arrhythmia screening success rates may be overestimated.

## Conclusions

Biatrial enlargement (BAE) is an independent predictor for reablation in atrial fibrillation patients and is associated with reablation in our follow up.

## Figures and Tables

**Figure 4:**
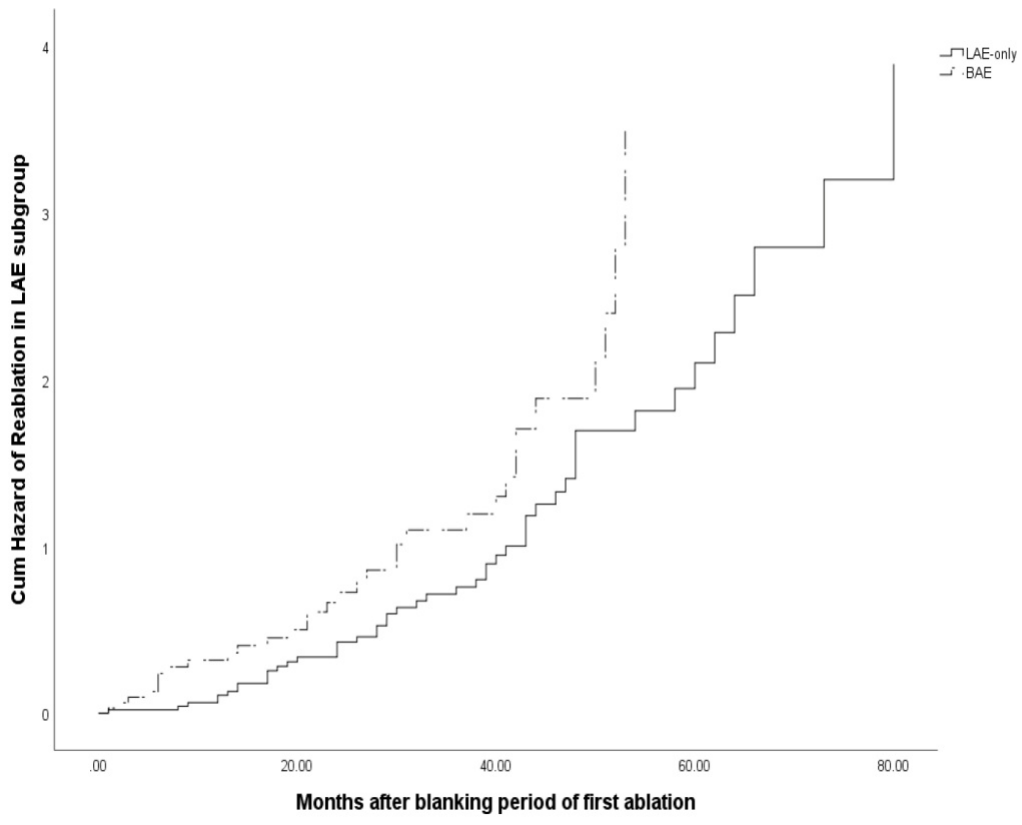
Cum hazard of reablation for subgroup analysis based on BAE in LAE group.

**Table 1:** Characteristics of the study group

Value	Total	PAF(n=361)	Non-PAF(n=158)	P
Age, years	65.39±10.65	65.60±10.77	64.91±10.39	0.493
Sex (female), n (%)	209(40.3)	147(40.7)	62(39.2)	0.771
Hypertension, n (%)	288(55.5)	202(56.0)	86(54.4)	0.774
Diabetes mellitus, n (%)	111(21.4)	80(22.2)	31(19.6)	0.280
Heart failure, n (%)	29(5.6)	12(3.3)	17(10.8)	0.001
CHADS2	1(0, 2)	1(0, 2)	1 (0, 2)	0.659
CHADS-VASC	2(1, 3)	2(1, 3)	2(1, 3)	0.911
Left atrium diameter, mm	41(36, 45)	38(35, 42)	44(41, 48)	0.000
Right atrium diameter, mm	36(33, 39)	35(32, 37)	39(37, 44)	0.000
LVEF, %	66(60, 70)	66(61, 70.5)	64.5(59, 68)	0.004
LVIDD, mm	50(47, 54)	50(47, 53)	52(47, 55)	0.042
Cardiac output, (L/s)	5.5(4.7, 6.6)	5.35(4.7, 6.1)	5.85(5, 7.1)	0.000
Mitral E peek, (cm/s)	88(70, 107)	80(67, 102)	101(85, 117.5)	0.000

LVEF: left ventricular ejection fraction. LVIDD: left ventricular internal diameter at end-diastole

**Table 2:** Clinical characters between BAE and Non-BAE

Value	Total	BAE(n=100)	Non-BAE(n=419)	P
Age, years	65.39±10.65	65.39±10.38	65.39±10.72	0.944
Sex (female), n (%)	209(40.3)	34(34.0)	175(41.8)	0.174
Hypertension, n (%)	288(55.5)	56(56.0)	232(55.4)	0.909
Diebetes mellitus, n (%)	111(21.4)	22(22.0)	89(21.2)	0.892
CAD, n(%)	86(16.6)	22(22.0)	64(15.3)	0.133
Heart failure, n (%)	29(5.6)	18(18.0)	11(2.6)	0.000
CHADS2	1(0, 2)	1(1, 2)	1(0, 2)	0.119
CHADS-VASC	2(1, 3)	2(1, 4)	2(1, 3)	0.219
Persistent AF, n(%)	158(30.4)	72(72.0)	86(20.5)	0.000
Left atrium diameter, mm	41(36, 45)	46(44, 49)	39(36, 43)	0.000
right atrium diameter, mm	36(33, 39)	44(42, 47)	35(33, 37)	0.000
RVIDD, mm	16(16, 18)	16(16, 19)	16(16, 17)	0.009
LVEF, %	66(60, 70)	63(55, 68)	66(61, 70)	0.000
LVIDD, mm	50(47, 54)	53(49, 56)	50(47, 53)	0.000
EDV,	122(104, 140)	135(112, 155)	119(103, 135)	0.000
Cardiac output, (L/s)	5.5(4.7, 6.6)	5.95(5, 7)	5.4(4.7, 6.4)	0.008
PVmax, (cm/s)	100(87, 111)	94.5(80, 108)	100(88, 112)	0.005
AVmax, (cm/s)	133(117, 152)	125(110, 141)	135(118, 154)	0.002
Mitral E peek, (cm/s)	88(70, 107)	100(82, 116)	85(69, 106)	0.000
Tricuspid E peek, (cm/s)	56(49, 64)	59(51, 69)	55(49, 63)	0.011
Procedure time, min	120(92.5, 150)	140(115, 180)	110(90, 140)	0.000
PVI+linear ablation, n (%)	273(52.6)	88(88.0)	185(44.2)	0.000
Cardioversion, n (%)	146(28.2)	68(68.0)	78(18.7)	0.000
Recurrence, n (%)	170(32.8)	52(52.0)	118(28.2)	0.000
Reablation, n(%)	117(22.5)	33(33.0)	84(20.0)	0.007

CAD: coronary artery disease. RVIDD: right ventricular internal diameter at end-diastole. LVEF: left ventricular ejection fraction. LVIDD: left ventricular internal diameter at end-diastole. EDV: end-diastole volume. PVmax: pulmonary artery maximum velocity. AVmax: aortic maximum velocity.

**Table 3:** Comparison between the two groups of patients with recurrence of AF

Value	Recur (n=170)	Reablation (n=117)	Drugs(n=53)	P
Age, years	65.39±10.65	65.82±9.33	69.49±8.49	0.016
Sex (female), n (%)	74(43.5)	50(42.7)	24(45.3)	0.442
Persistent AF, n(%)	76(44.7)	47(40.2)	24(54.7)	0.055
Hypertension, n (%)	98(57.6)	70(59.8)	28(52.8)	0.245
Diabetes mellitus, n (%)	35(20.6)	20(17.1)	15(28.3)	0.073
CAD, n(%)	35(20.6)	19(16.2)	16(30.2)	0.032
Heart failure, n (%)	11(6.5)	6(5.1)	5(9.4)	0.231
CHADS2	1(0, 2)	1(0, 2)	1 (0.5, 2)	0.106
CHADS-VASC	2(1, 3.75)	2(1, 3)	2(1, 3)	0.016
Left atrium diameter, mm	42(39, 46)	42(39, 46)	44(39.5, 46)	0.566
Right atrium diameter, mm	37.5(34, 42)	37(34, 41)	39(35, 44.5)	0.141
LVEF, %	65(59, 70)	65(59, 70)	65(58, 69)	0.976
LVIDD, mm	51(47, 54)	51(47, 54)	50(46, 55)	0.276
Cardiac output, (L/s)	5.5(4.8, 6.6)	5.4(4.9, 6.6)	5.7(4.5,6.6)	0.784
Mitral E peek, (cm/s)	96.5(75, 111)	94(76, 110)	101(75, 122)	0.190

CAD: coronary artery disease. LVEF: left ventricular ejection fraction. LVIDD: left ventricular internal diameter at end-diastole.

**Table 4:** Correlations of reablation with clinical characters

	r	P		r	P
Age	0.21	0.626	LAD	0.150	0.001**
Sex	0.27	0.538	RAD	0.092	0.036*
Persistent AF	0.114	0.009**	LVEF	-0.022	0.625
Hypertension	0.047	0.284	LVIDD	0.029	-0.509
Diabetes mellitus	-0.056	0.199	EDV	0.033	0.448
CAD	-0.005	0.913	ME	0.092	0.036*
Heart failure	-0.011	0.806	TE	0.034	0.439
BAE	0.122	0.005**			

CAD: coronary artery disease. HF: heart failure. LAD: left atrium diameter. RAD: right atrium diameter. LVEF: left ventricular ejection fraction. LVIDD: left ventricular internal diameter at end-diastole. EDV: end-diastole volume. ME: mitral E peak. TE: tricuspid E peak. BAE: biatrial enlargement. ** P<0.01. * P<0.05.

**Table 5:** Multivariate Cox regression analysis of reablation

	HR	95%C.I	P
Age	1.001	0.978-1.024	0.942
Sex	1.067	0.702-1.622	0.763
Persistent AF	0.746	0.474-1.174	0.205
HT	1.055	0.681-1.636	0.809
DM	1.371	0.790-2.377	0.262
CAD	0.762	0.443-1.309	0.325
LVEF	1.010	0.980-1.040	0.525
EDV	0.998	0.991-1.005	0.603
Mitral E peek	1.004	0.997-1.011	0.288
BAE	1.755	1.153-2.670	0.009

**Table 6:** Subgroup analysis based on BAE in patients with LAE

Value	BAE(n=100)	LAE only(n=187)	P
Heart failure, n (%)	18(18)	9(4.8)	0.000
Persistent AF, n(%)	72(72)	68(36.4)	0.000
Left atrium diameter, mm	46(44, 49)	43(41, 46)	0.000
Right atrium diameter, mm	44(42, 47)	37(35, 38)	0.000
RVEDD, mm	16(16, 19)	16(16, 17)	0.002
LVEF, %	63(55, 68)	66(60, 70)	0.001
LVEDD, mm	53(49, 56)	52(48, 54)	0.121
EDV, mL	135(112, 155)	128(111, 146)	0.110
Tricuspid E peek, (cm/s)	59(51, 69)	55(48, 63)	0.018
Procedure time, min	140(115, 180)	120(100, 150)	0.001
PVI+linear ablation, n (%)	88(88)	105(56.1)	0.000
Electrical Cardioversion, n (%)	68(68)	61(32.8)	0.000
Recurrence, n (%)	52(52)	70(37.4)	0.000
Reablation, n (%)	33(33)	49(26.2)	0.001

Multivariate Cox regression analysis demonstrated that BAE (HR 1.620, 95%CI 1.020-2.574, P=0.041) was an independent predictor for reablation among the patients with LAE. See Table 7. And, BAE was also associated with more reablation (Log rank P=0.026) in these patients, see Figure 4.

**Table 7:** Multivariate Cox regression analysis of reablation among the patients with LAE

	HR	95%C.I	P
Age	1.006	0.977-1.036	0.700
Sex	0.860	0.504-1.467	0.579
Persistent AF	0.690	0.413-1.152	0.156
HT	1.129	0.668-1.908	0.650
DM	1.156	0.521-2.564	0.722
CAD	0.785	0.396-1.555	0.488
LVEF	0.991	0.961-1.023	0.574
EDV	0.999	0.992-1.007	0.876
Mitral E peek	1.003	0.994-1.011	0.513
BAE	1.620	1.020-2.574	0.041
